# Investigating the Ability of Growth Models to Predict In Situ *Vibrio* spp. Abundances

**DOI:** 10.3390/microorganisms10091765

**Published:** 2022-08-31

**Authors:** Marija Purgar, Damir Kapetanović, Sunčana Geček, Nina Marn, Ines Haberle, Branimir K. Hackenberger, Ana Gavrilović, Jadranka Pečar Ilić, Domagoj K. Hackenberger, Tamara Djerdj, Bruno Ćaleta, Tin Klanjscek

**Affiliations:** 1 Ruđer Bošković Institute, 10000 Zagreb, Croatia; 2 School of Biological Sciences, The University of Western Australia, Crawley, WA 6009, Australia; 3 Department of Biology, Josip Juraj Strossmayer University of Osijek, 31000 Osijek, Croatia; 4 Faculty of Agriculture, University of Zagreb, 10000 Zagreb, Croatia

**Keywords:** mechanistic modeling, primary and secondary growth models overview, comprehensive datasets, bacterial growth

## Abstract

*Vibrio* spp. have an important role in biogeochemical cycles; some species are disease agents for aquatic animals and/or humans. Predicting population dynamics of *Vibrio* spp. in natural environments is crucial to predicting how the future conditions will affect the dynamics of these bacteria. The majority of existing *Vibrio* spp. population growth models were developed in controlled environments, and their applicability to natural environments is unknown. We collected all available functional models from the literature, and distilled them into 28 variants using unified nomenclature. Next, we assessed their ability to predict *Vibrio* spp. abundance using two new and five already published longitudinal datasets on *Vibrio* abundance in four different habitat types. Results demonstrate that, while the models were able to predict *Vibrio* spp. abundance to an extent, the predictions were not reliable. Models often underperformed, especially in environments under significant anthropogenic influence such as aquaculture and urban coastal habitats. We discuss implications and limitations of our analysis, and suggest research priorities; in particular, we advocate for measuring and modeling organic matter.

## 1. Introduction

*Vibrio* spp. are naturally occurring aquatic bacteria, highly adaptive and freely associated with a variety of biotic and abiotic surfaces including water, sediment, fish, shellfish, algae, and zooplankton. *Vibrio* spp. comprise a minor portion of the total microbial population, and around 1 percent of the total bacterioplankton in coastal waters [1]. Despite their relatively low abundance, *Vibrio* species are one of key constituents of aquatic heterotrophic bacterial groups [2].

Aquatic heterotrophic microorganisms have an important role in the mineralization of organic matter, and the variations in abundance, community structure, and activities of heterotrophic microbial communities affect both the biotic and the abiotic components of aquatic environments. *Vibrio* spp. participate in biogeochemical processes by utilizing a variety of substrates and mineralization of organic matter, thus directly contributing to the recycling of carbon, nitrogen, and organic matter in the aquatic environment [1,3,4]. Alongside their role in the abiotic cycles, some >140 described species from the genus *Vibrio* can have a strong biotic impact, and consequently pose severe health risks and economic losses. A well-known example is *Vibrio cholerae*, which causes cholera—a global threat to public health with about four million cases of infection every year, leading to over 100,000 deaths [5]. The rise of noncholera *Vibrio* species (*V. parahaemolyticus*, *V. alginolyticus*, and *V. vulnificus*) can cause other potentially lethal infections (vibriosis) in humans [2], and some of the well-known *Vibrio* pathogens (e.g., *V. anguillarum*, *V. harveyi*, *V. vulnificus*, *V. salmonicida*) are harmful to aquatic (marine) organisms; these species induce vibriosis in fish and other marine species, which results in massive economic losses for the aquaculture industry worldwide.

Outbreaks of vibriosis naturally arise mainly with fluctuations in the physicochemical properties of water such as temperature, salinity, dissolved oxygen, and nutrient pulses (e.g., phytoplankton blooms and dust storms). Fluctuations are supported by the fast response of *Vibrio* spp. to favorable environmental conditions [6,7]. The ongoing climate change adds complexity to the environmental patterns, as it induces shifts in the marine environments by increasing temperature, altering nutrient loads, shifting precipitation patterns, and acidifying the ocean [8,9,10]. This, in turn, affects the *Vibrio* spp. abundance and alters the distribution of infectious diseases such as vibriosis [11]. Climate change can also initiate the lengthening of the seasonal period of maximal *Vibrio* concentrations and broaden the areas permitting the survival of these pathogens [12]. Therefore, in order to develop informed strategies to minimize vibriosis outbreaks and prevent potential health risks and aquaculture economic losses, it is crucial to take both *Vibrio* spp. dynamics and the environment into account.

Mathematical modeling, analysis, and simulations provide useful insights into *Vibrio* spp. abundance in various natural or anthropogenic systems. They help in developing management strategies, and advance the knowledge of changes in the microbial communities in aquatic environments. Accurate predictions can also advance the decision-making processes of aquaculture and estimation of costs, as well as the enactment of legislation in food safety and water research, which are two major areas in applied microbiology [13,14]. The main approach to modeling the *Vibrio* spp. is based on empirical techniques, where models are analyzed from statistical, numerical, and computational points of view, such as goodness of fit or standard errors of the estimated parameters. Mathematical models in food safety research (e.g., [15,16,17,18]) are generally categorized into primary, secondary, and tertiary models [19].

Microbial growth curves show the number of living cells as a function of time [17]. Primary models typically describe isothermal growth, i.e., growth as a function of time at a constant temperature. Under constant (laboratory) conditions, bacterial growth is characterized by a sigmoid curve where the dependent variable is the logarithm of the viable cell concentration [20]. The slope of a sigmoid curve at a given time provides the instantaneous specific growth rate, which can be considered as the “cells’ per capita rate of division” [21]. The other two essential parameters of primary models are the maximum specific growth rate at the inflexion point of the sigmoid curve, and the length of the lag phase.

Secondary mathematical models describe the functional dependence of microbial growth on external factors such as temperature or pH [17]. Commonly used secondary models are the square-root model (Ratkowsky model [22]) and the Arrhenius-based model [23], which provide satisfactory descriptions of the dependence of growth on temperature, pH, or other factors [24]. Finally, tertiary mathematical models are software packages that combine primary and/or secondary models and often add a user interface [24].

Generally, all described models are useful, but they are not always applicable. The described mathematical models can be used for description and prediction of *Vibrio* spp. abundance only under certain (known and tested) conditions. The limitations stem from model formulation: while the equations provide satisfactory descriptions of *Vibrio* growth and its dependence on, e.g., temperature or pH, they usually are not subject to mechanistic interpretation [24], and therefore should not be used for explanatory purposes or predictions under untested conditions. The issue of the applicability and usability of a model becomes important when we wish to apply a model to understand or predict *Vibrio* spp. dynamics in situ. For data collected in situ, exact conditions of data collection are unknown, intervals between data collection are long and/or irregular, and environmental conditions are rarely constant.

We aimed to (i) identify and systematize primary and secondary *Vibrio* spp. growth models, and (ii) to analyze and validate the models by applying them to different sets of available data. We reviewed growth models of *Vibrio* spp. using examples from research in food safety and water research, and validated them on several sets of field data. Then, we analyzed whether the model(s) can be used to capture in situ *Vibrio* spp. abundances, with  an emphasis on differences between the marine habitat types.

## 2. Materials and Methods

**Figure 1:**
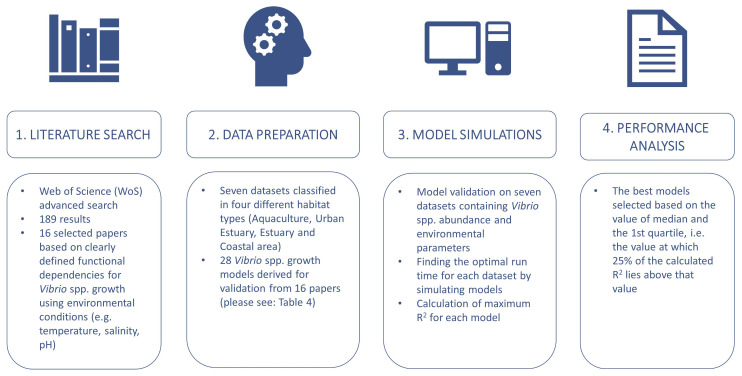
The methodological approach to model analysis. We performed a literature search to find all *Vibrio* spp. growth models and all available datasets suitable for comparison. We found five published datasets, and included two of our own collected through projects AqADAPT and AQUAHEALTH of the Croatian Science Foundation (HRZZ).

Our methodological approach can be divided into four main steps: (1) a literature search, (2) data preparation, (3) model simulations, and (4) performance analysis. The methodology overview is graphically presented in Figure 1.

### 2.1. Literature Search and Model Synthesis

The literature search was conducted using the Web of Science (WoS) advanced search in April 2022. The search string was defined based on keywords and Boolean and adjacency operators, and was searched for in Abstracts (Field Tag “AB”). The search string was as follows: AB = (((vibrio*) AND (growth OR abundance) AND (temperature OR salinity OR “pH” OR “COD” OR “organic matter” OR nutrient*) AND (model*))). We obtained 189 results based on our search, which was restricted to the English language. For  a detailed description of our literature review, please see Appendix A. We selected 16 papers with *Vibrio* spp. growth models based on clearly defined functional dependencies for using environmental conditions (e.g., temperature, salinity, pH). We did not include models derived purely by regression or similar statistical means because those are not modular, and cannot be differentiated into primary and secondary. From the 16 papers containing growth models, we extracted, systematized, and classified explicit formulations for primary (Table 1) and secondary growth models (Table 2), using parameters listed in (Table 3) and thus arriving at 28 unique *Vibrio* spp. growth models for further analysis (Table 4).

### 2.2. Data Preparation

An additional literature search identified five datasets suitable for model validation; hence, a total of seven datasets were available for analysis (Table 5) once our two previously unpublished datasets were added.

The previously unpublished datasets, AqADAPT [54] and AQUAHEALTH [55], contain observed values for *Vibrio* spp. abundance and environmental parameters from the Adriatic Sea. Sampling was conducted in three floating-cage fish farms in the northern, middle, and southern Adriatic Sea (Croatia), where European sea bass (*Dicentrarchus labrax*) and sea bream (*Sparus aurata*) are cultured. The farms in the northern and central Adriatic are located in the semi-open sea at depths of about 49 m and 22 m, respectively. The fish farm in the southern Adriatic is located in the outer part of the Mali Ston Bay at a depth of 18 m. The Mali Ston Bay is occasionally strongly influenced by the (freshwater) Neretva River. Periodic use of antibiotics is possible on all three fish farms, and this would have affected the *Vibrio* spp. abundance. However, no specific data on antibiotics use are available. We classified these datasets into aquaculture habitat type. Dataset AqADAPT [54] was labeled as AQC1 and dataset AQUAHEALTH [55] was labeled as AQC2.

Bullington et al. [56] and Steward et al. [57] published *Vibrio* spp. abundance and environmental parameters from the Ala Wai Canal in urban Honolulu, Hawaii, on the island of O’ahu. The 3.1 km-long engineered waterway operates as a tidally influenced estuary with freshwater input from a watershed that covers 42.4 km^2^ via the Manoa and Palolo streams, which merge to form the Manoa–Palolo Stream prior to entering the canal, and the Makiki Stream, all of which run through urban areas before reaching the canal. Consequently, the streams are contaminated with a variety of anthropogenic substances, and their convergence in the Ala Wai Canal has contributed to its pollution and eutrophication [57]. We classified datasets from this area as the urban estuary habitat type due to the strong anthropogenic influence. The dataset by Bullington et al. [56] was labeled as URB1 and the dataset by Steward et al. [57] was labeled as URB2.

Froelich et al. [58] gathered data from the Neuse River Estuary in Eastern North Carolina (USA). The Neuse River Estuary, located in Eastern NC (USA), is a well-described, lagoonal estuary, with wind-driven mixing characteristics and minimal tidal influence due to the protection offered by the proximal Pamlico Sound. Being broad and shallow (generally less than 3 m in depth), the estuary flow and mixing are dominated by wind and river input. This dataset was classified as an estuary habitat and labeled as EST1.

Urquhart et al. [59] collected data from the Great Bay Estuary, New Hampshire (USA). The Great Bay Estuary (GBE) extends inland from the mouth of the Piscataqua River near Kittery, ME, through Little Bay and eventually into the Great Bay (25 km). The GBE has deep, narrow channels with strong tidal currents, and wide, shallow mudflats. The physical transport regime of the GBE follows the classical estuarine circulation model for drowned river valley estuaries [59]. This dataset was also classified as an estuary habitat and labeled EST2.

Williams et al. [60] presented data from five sites along the Eastern North Carolina coast (USA). Locations were as follows: Harlowe Creek, South River, North River, Hoop Pole Creek, and Jumping Run Creek. These sites were chosen to represent the range of high- and low-salinity environments, some of which experience large salinity fluctuations, while others have very small salinity fluctuations (for more information, please refer to the original manuscript [60]). We classified this dataset as a coastal area, and labeled it COAST.

From the given datasets, we selected variable *Vibrio* abundance and the following environmental parameters: temperature, salinity, and pH. We excluded all of the missing values from datasets and logarithmically transformed *Vibrio* abundance greater than 0 (log10 + 1 in datasets AQC1, AQC2, and COAST, log10 in datasets URB1 and URB2). Datasets EST1 and EST2 already contained logarithmically transformed values. The analytical code can be found in the R script named data_preparation. Results of the dataset preparation are summarized in Table 5.

### 2.3. Model Simulations

Herein, we showcase a model simulation approach aiming to calculate *Vibrio* spp. abundance based on primary and secondary models accounting for environmental parameters (temperature, salinity, and pH). All collected models were developed for controlled environments (i.e., laboratories), where *Vibrio* spp. growth was monitored at regular, short time intervals. Such data lend themselves to time series modeling, where abundance is plotted against time. In contrast, in situ data are typically irregularly collected from variable abiotic microenvironments, at longer time intervals, and with the noise typically inherent to field measurements. Such data could not be modeled as a time series, but had to be modeled as independent abundances—each data point was considered to be a result of bacterial growth that started some time ago.

The model had to be simulated for the time of growth, but determining how long ago the growth started was a challenge which we needed to overcome in order to select the (optimal) model run time for a dataset. Note that having an independent run time for each data point would create an option to fit each data point exactly by choosing the perfect time, thus defeating the whole point of modeling the bacterial dynamics. To minimize the bias introduced by our choice of the model run time, we determined a (run) time that gives the best result for each model-and-dataset combination. Optimal run time is then the simulation duration that produces the best match between the model predictions and the observations.

We used default parameter values (Appendix B, Table A1) listed in their respective references for each of the 28 models (Table 4). First, the values of the specific growth rate and lag time were calculated, which were then used in the primary models: modified logistic, Barayni, Gompertz, modified Gompertz, three-phase linear, Huang, no-lag phase and met exponential models. These primary models adjust the specific growth rate and lag time using one or more of the secondary models, as described in the original literature and summarized in Table 4. The environmental parameters considered in the literature for modeling *Vibrio* spp. growth in dynamic conditions were: temperature, salinity, and pH (Table 4). Secondary models sometimes use water activity or NaCl concentration instead of salinity. As all datasets contained information on salinity, water activity or NaCl concentration were calculated from salinity (Table 5) (for more details, please refer to Appendix B). To determine the optimal run time, we simulated *Vibrio* spp. growth in a selected time range from 1 to 600 hours, and selected the run time that produced the best fit to the data.

### 2.4. Model Performance

We used the coefficient of determination ($R^{2}$) to evaluate the ability of models to describe the observed experimental data:

(21) $$R^{2}=1-\frac{\sum_{i=1}^{n}{\left(y_{i}-\hat{y_{i}}\right)}^{2}}{\sum_{i=1}^{n}{\left(y_{i}-\bar{y}\right)}^{2}},$$

where $y_{i}$ is the actual value in the dataset, $\hat{y_{i}}$ is the corresponding predicted value, $\bar{y}$ is the mean value of the dataset, and *n* is the sample size.

To determine applicability (i.e., model generality) of a specific model to a specific dataset, we compared $R^{2}$ values calculated for all models for that specific dataset, and then selected those models for which the dataset-specific $R^{2}$ value was higher than the overall median of all $R^{2}$ values for all models and all datasets. For example, if the median overall goodness of fit of all models to all datasets was $R^{2}=0.30$, all models with $R^{2}>0.30$ for dataset 1 would be marked as capturing dataset 1. We then tested the robustness of the results by looking at a more stringent requirement, where we marked a certain model as capturing a dataset only if its $R^{2}$ for that dataset was in the top 25% (1st quartile) of the values for all models and all datasets.

Robust ANOVA based on trimmed means [61,62] was used to test the difference in model performance (obtained $R^{2}$) between different habitat types (aquaculture, urban estuary, estuary, and coastal area). Robust ANOVA was used to overcome the problems associated with deviations from homoscedasticity/normality and to reduce the influence of outliers observed in the data. Post hoc tests were also performed in the robust WRS2 environment [61], where p-values were adjusted for multiple testing using the Benjamini–Hochberg (BH) method.

The model analysis and simulations were performed in RStudio Integrated Development Environment, Version 4.1.2 [63] using the packages: tidyverse [64], caret [65], Metrics [66], SciViews [67], data.table [68], readxl [69] and xlsx [70]. The exact analytic code can be found at Zenodo [71]. We visualized results using the package ggplot2 [72].

## 3. Results

### 3.1. *Vibrio* spp. Growth Models

*Vibrio* spp. growth models identified by the literature review could be partitioned into 12 primary (Table 1) and 8 secondary (Table 2) models, using the parameters listed in Table 3. In total, we identified 29 models for *Vibrio* spp. growth in a dynamic environment, but we could not find parameter values for Fujikawa et al. [50]. Therefore, we further analyzed only the remaining 28 models (Table 4), using the parameters listed in Table A1.

The model summaries (Table 4) provide an overview of the models used when studying *Vibrio* spp. growth in a dynamic environment. The main findings were as follows:

- Baranyi and modified Gompertz are the most commonly used primary models for describing *Vibrio* spp. growth over time.
- Square root and Arrhenius-based models are the most frequently applied secondary models for *Vibrio* spp. growth in dynamic conditions.
- *V. cholerae*, *V. parahaemolyticus*, *V. harveyi*, and *V. vulnificus* are the species used most often as modeling organisms.
- *Vibrio* growth was monitored in/on various substrates (free water column, within organisms, in broth substrates, etc.), under different temperature, salinity, and pH conditions. This implies that the aquatic environments and organisms (marine and freshwater), as well as food and water health and safety, are the key areas of research and concern.
- Temperature was the prevailing environmental parameter used in secondary models, implying a strong effect of temperature on *Vibrio* spp. abundance. The effect of temperature on the primary model parameters (growth rate and lag time) was most often modeled by the square root or the Arrhenius-based model.

### 3.2. *Vibrio* In Situ Datasets

We summarize the dataset classification and characteristics of the seven datasets in Table 5, including the number of *Vibrio* spp. abundance data, and range and type of environmental variables used in model validation (temperature, salinity, pH). Datasets URB2 and COAST do not contain information on pH, so simulations of Model 6 (Table 4) were not possible for those datasets.

### 3.3. Model Performance

**Figure 2:**
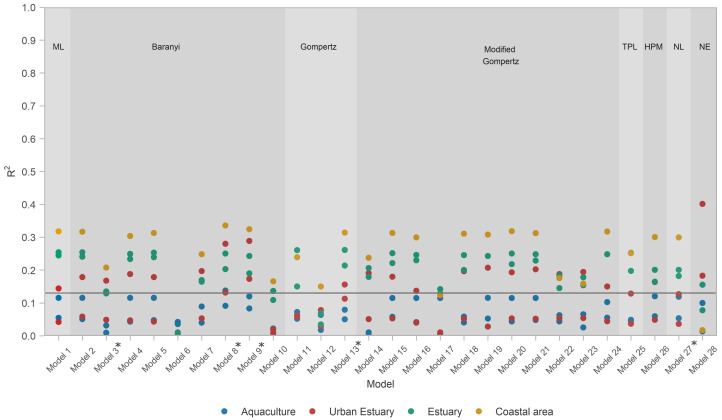
*R*^2^ calculated for all models and for each dataset. Horizontal line depicts the median $R^{2}=0.13$ used to evaluate model performance. Primary models are labeled as follows: ML—modified logistic, Baranyi, Gompertz, modified Gompertz, TPL—three-phase linear, HPM—Huang primary, NL—no-lag and NE—net exponential. Star (*) signifies models that had an evaluation issue with some of the data points in some of the datasets (details in Appendix D).

**Figure 3:**
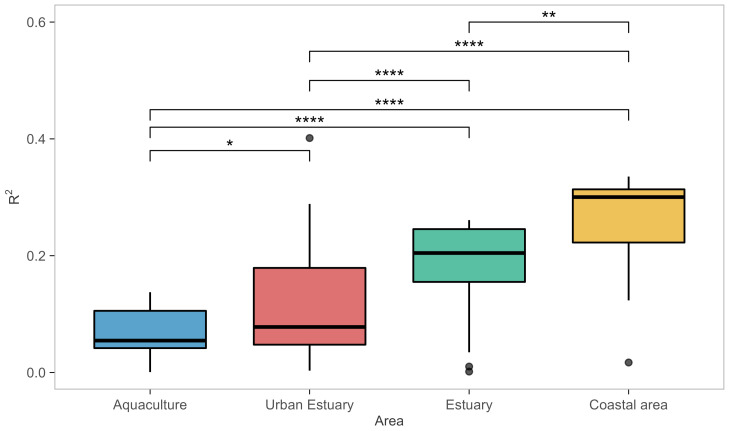
Boxplot of model performance measured as $R^{2}$ in different habitat types (as classified in Table 5). Model performance significantly differed between habitat types (robust ANOVA, F(3,75.561) = 49.9, *p* < 0.001, effect size ξ = 0.77, confidence interval CI(ξ) = [0.68, 0.84]; Table A2). Significance codes are as follows: p≤0 ‘****’, p<0.01 ‘**’, and p<0.5 ‘*’.

The ability of models to describe the data greatly varied between models and datasets (Figure 2). The calculated $R^{2}$ values ranged from <0.001 (Model 10 for dataset AQC1) to 0.40 (Model 28, dataset URB2), with the overall median value of all models for all datasets $R^{2}=0.13$. $R^{2}$ values also significantly differed between habitat types (Figure 3, Table A2) in all pairwise comparisons (post hoc tests *p* < 0.017; see details in Table A3). Therefore, $R^{2}$ values suggest the models performed best for coastal areas, followed by estuaries, urban estuaries, and aquaculture habitats. Comparing $R^{2}$ values provides performance estimates of a particular model on a particular dataset, but does not provide information on generality.

**Figure 4:**
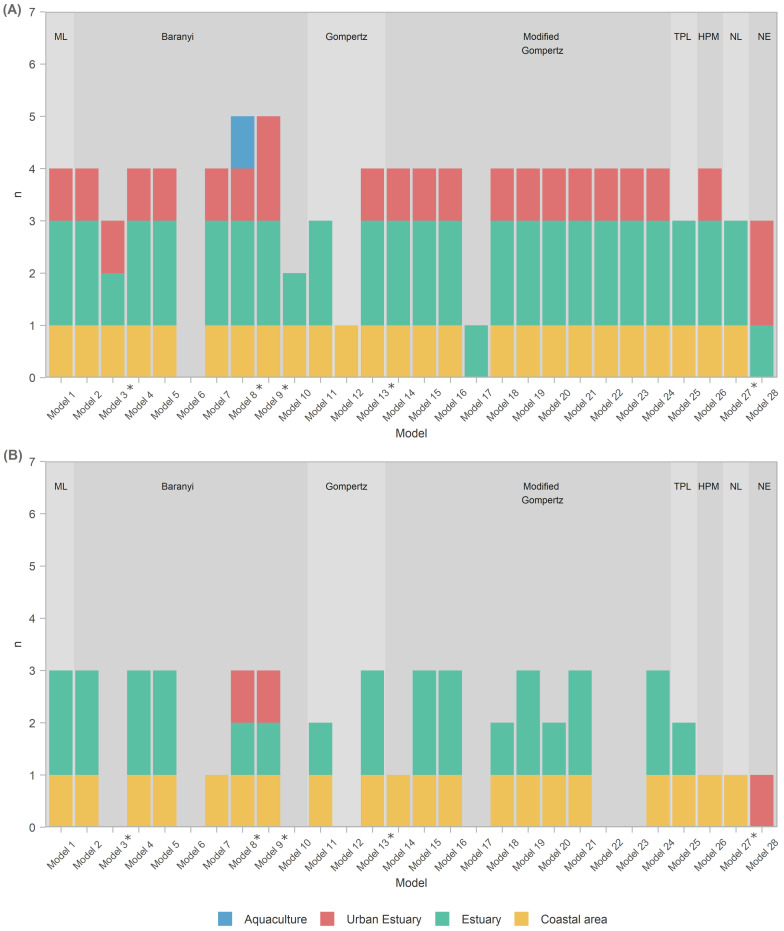
Model generality. Stacked bar chart used for graphical representation of model’s applicability on the dataset from a specific habitat: colors represent habitats (see the legend), and stars (*) denote models that exhibit the evaluation issue with some of the data points in some of the datasets (details in Appendix D). Values on y axis denote the frequency of occurrence of a particular model whose R^2^ value is above the median ($R^{2}>0.13$; Panel **A**), and in the first quartile ($R^{2}>0.22$; Panel **B**). Primary models are labeled as follows: ML—modified logistic, Baranyi, Gompertz, modified Gompertz, TPL—three-phase linear, HPM—Huang primary, NL—no-lag and NE—net exponential. Star (*) signifies models that had an evaluation issue with some of the data points in some of the datasets (details in Appendix D). Note that there is only one coastal area dataset, while other habitats have two datasets each. Hence, scoring a single occurrence of the coastal area habitat represents a 100% success rate, while scoring the same in any other habitat represents a success rate of 50%.

Model generality analysis (Figure 4A) shows rankings above the median ($R^{2}>0.13$) where:

- All models except Model 6 (Baranyi with polinomial pH and salinity secondary models) were able to score above average at least in some habitats, i.e., capture those habitats. Incidentally, Model 6 was the only one *not* applying a temperature correction
- A total of 93% of models captured the coastal area habitat.
- A total of 93% of all models captured estuary habitat, but only 85% of those (i.e., 79% of all models) captured both estuary datasets; 26 models captured EST1, with 22 of them capturing EST2 as well.
- A total of 75% of models captured an urban habitat, but only two (Models 9 and 28) captured both urban habitat datasets; URB1 was captured by all 20 of them; only 3 models managed to capture URB2.
- Only the Baranyi-type model (Model 8, with temperature and salinity secondary models) captured the AQC1 aquaculture habitat.
- The model with the highest $R^{2}$ values (Model 28—net exponential, for urban estuary URB2) had low generality, as it captured datasets from only two out of four habitats.
- Of the models that performed well for at least one habitat type, Model 17 had lowest generality as it captured only one EST1 dataset.

Selecting for better capability by scoring only performances within the first quartile ($R^{2}>0.22$, Figure 4B) shows that:

- ≈A total of 72% of the analyzed models had an exceptional ability to capture datasets from the coastal area habitat.
- The ability of models to capture/perform well for estuarine habitats was severely diminished, with only 16/28 (57% of the models) capturing one of the two estuary datasets, and only 10 models (36%) capturing both.
- None of the models were able to capture for the aquaculture habitat datasets, and only three (Models 8, 9, and 28) captured the urban estuary habitat.
- Model 28 seemed even more specialized, as it captured a single urban estuary (URB2)
- Most prolific Baranyi models (Models 8 and 9) remained so by capturing datasets from three habitat types, albeit only a single dataset from each.

## 4. Discussion

Our work adds to the growing body of knowledge from the past few decades that helped refine a general understanding of the ecology of *Vibrio* spp. We (i) summarized dynamics and unified nomenclature for all published functional models we could find (12 primary and 8 secondary), (ii) added two datasets to the existing five longitudinal datasets on *Vibrio* spp. and their environments, and (iii) used the datasets to asses the ability of existing models to capture *Vibrio* spp. abundances in four different habitat types.

There are no clear winners between the 28 investigated models. Generally, the $R^{2}$ values were not particularly large (≤0.40), but the values can be considered acceptable given the difficulty of the task, in particular the multitude of potential factors affecting both the environment and the *Vibrio* populations.

Baranyi-based models (Models 2 to 10 in Table 4) seemed to capture the widest variety of habitats well, with Models 8 and 9 leading the way in diversity. Although both models had a similar $R^{2}$ for the AQC1 dataset (0.14 and 0.12, respectively), Model 8 was the only one that crossed the median threshold and therefore captured an aquaculture dataset as an above-average performer. Notably, Models 8 and 9 are the only Baranyi-based models that included both temperature and salinity secondary models. Model 28 (net exponential) also included both factors and performed quite well, giving the highest overall $R^{2}$ (0.40 for the URB2 dataset).

Inclusion of temperature and salinity, however, does not guarantee success. All three Gompertz-based models included both factors, but all three underperformed. This would signal Gompertz-based models should be used with caution. Modified Gompertz-based models (Models 14–24), however, seem to lag only slightly behind the best.

While it may be tempting to proclaim Baranyi-based models as the most versatile, they do have significant drawbacks. First, both Models 8 and 9 had issues with simulations: their fast growth rate sometimes caused very large predictions (see Appendix D). While this has not been a problem for the optimal run time of Model 8, Model 9 did lose up to 3% of data in the evaluation. These issues may not be consequential in the current assessment, but may become so in new datasets.

**Figure 5:**
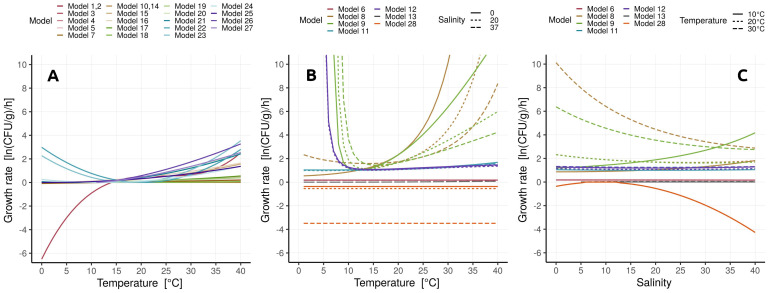
Relationships between growth rate and environmental variables as predicted by secondary models. The models in panel (**A**) include only a temperature correction. Panel (**B**) shows dependence of growth rate on temperature for three salinity levels. Panel (**C**) shows dependence of growth rate on salinity for three temperatures. A pH value of 8.1 was assumed for Model 6. Model 28 had a flat temperature response because it used the default parameter value [49] that minimized temperature correction, θ=1; increasing θ would increase the temperature dependence.

Second, the secondary models accounting for salinity in Models 8 and 9 yielded unexpected growth rate patterns. For example, at moderate and high salinities, growth rates could be extremely high at the ends of the environmental temperature range (e.g., 3.18 ln(CFU/g)/h for salinity of 20 and temperature of 27 °C, Figure 5, Panel B). Likewise, at 10 °C, the growth rate of Model 8 was moderate and slightly *increased* with salinity; at 30 °C, however, the rate was extremely high for low salinity, and rapidly *decreased* with salinity. This may be plausible as abundance of *Vibrio* spp. increased with water temperatures during periods of reduced salinity [73], but we recommend caution when utilizing Baranyi-based models in highly variable environments.

Overall, predictions for the coastal area and estuary datasets seemed to be more robust than those for urban estuaries and aquaculture (Figure 4, Panel B). We hypothesize this may be due to higher levels of anthropogenic disturbance in aquaculture and urban estuaries, in particular potentially high inputs of organic matter. *Vibrio* spp., as a prototypical copiotroph, dominates in nutrient-rich environments [74]. They exhibit a feast-and-famine lifestyle and swim to colonize sporadic, nutrient-rich patches and particles [75]. Therefore, we think that organic matter has an important role in determining *Vibrio* spp. abundance, especially in these types of habitats.

Our study has some limitations. First, we treated all *Vibrio* spp. the same. While justified in the context of a wide assessment such as ours, *Vibrio* species clearly differ and could potentially exhibit significantly different dynamics, especially since different species favor different environmental conditions. These differences could become important as environmental conditions change, and the *Vibrio* community could shift towards disease-causing species. Unfortunately, current datasets and available models do not allow for a deeper investigation of the issue.

Second, we chose the simulation time that minimized $R^{2}$, but relied on a single simulation time for each dataset. In principle, a different simulation time could be chosen for each data point. Such an approach would, however, in effect result in fitting each value by choosing simulation time that gives a desired result, thus defeating the purpose of modeling. There could, nonetheless, be some value in exploring functional dependencies of the simulation time on various environmental factors (temperature in particular), but additional research would be required to suggest a particular form of such a function.

Third, we set out to investigate *published* parameters and models with well-defined functional forms. Perhaps a different set of parameters and/or combinations of models could have described the datasets better. We have made a first step towards such research by systematizing primary and secondary models, parameters, and available—including two previously unpublished—datasets. Alternatively, *statistical* models could possibly be re-fitted to better capture the datasets. Such statistical approaches may be appropriate in some cases, e.g., when aiming to inter- or extrapolate data from a single area.

Clearly, additional research is needed for developing a growth model capable of predicting in situ *Vibrio* spp. abundances in natural environments. We suggest further development of secondary models should be one priority; dynamics of secondary models should be well-defined for the whole range of expected environmental factors, especially temperature and salinity. Modeling could also benefit from research on additional environmental factors such as organic matter, which has been shown to affect *Vibrio* growth [56]. Given the role of *Vibrio* spp. in aquatic environments, it is surprising that less than half datasets include organic matter measurements. We suggest that future modeling development should include organic matter, especially when sampling to ensure measurement of organic matter.

Perhaps a development of a third generation of models based on big data and deep learning could also work in synergy with mechanistic modeling to improve our ability to predict *Vibrio* spp. dynamics in a changing environment. Improved models could then enhance predictive frameworks, e.g., by replacing the basic ecological niche approach to estimating *Vibrio* presence used in exploration of future risk scenarios by Trinanes and Martinez-Urtaza [76].

In conclusion, none of the investigated models provide a complete solution: Baranyi-based models might be the most versatile, but other models (e.g., net exponential) may provide a better fit for a particular cause. Therefore, the choice of the model should be at least in part guided by the type of the environment and expected ranges of environmental factors; if many data lie outside of the well-described range of a particular secondary model (see Figure 5), perhaps a different model should be tried instead. Our summary and systematization (Table 1, Table 2, Table 3, Table 4 and Table 5) provide currently available primary and secondary models, and data, which can be used as a toolbox for model creation and testing.

## Figures and Tables

**Table 1:** Systematized equations for *Vibrio* spp. primary growth models. Of the 12 models listed in this table, one (new logistic model) did not have parameters listed, so only the remaining 11 were used in further analysis. The reference in the column “Model” is the original paper containing the equation. The column “Article” lists all published articles that used the given primary models.

Model	Equation	Article
Modified logistic [25]	(1) $Y\left(t\right)=\frac{A}{\left\{1+\exp\left[\frac{4\cdot \mu_{\max}}{A}\left(\lambda -t\right)+2\right]\right\}}$	[26,27,28,29]
Baranyi [30]	(2) $$\left\{\begin{array}{c} Y\left(t\right)=Y_{0}+\mu_{\max}A\left(t\right)-\ln\left[1+\frac{\exp(\mu_{\max}A\left(t\right))-1}{\exp(Y_{\max}-Y_{0})}\right]\\ A(t)=t+\frac{1}{\mu_{\max}}\ln\left[\exp\left(-\mu_{\max}t\right)+\exp\left(-\mu_{\max}\lambda \right)-\exp\left(-\mu_{\max}t-\mu_{\max}\lambda \right)\right] \end{array}\right.$$	[26,28,29,31,32,33,34,35,36,37]
Gompertz [25]	(3) $Y\left(t\right)=Y_{0}+A\left(e^{\left(-e^{-B\left(t-D\right)}\right)}\right)$	[37,38,39]
Modified Gompertz [25]	(4) $\;Y\left(t\right)=Y_{0}+A\exp\left\{-\exp\left[\frac{\mu_{\max}\cdot e}{A}\left(\lambda -t\right)+1\right]\right\}$	[28,29,31,40,41,42]
Weibull [43]	(5) $Y\left(t\right)=Y_{0}-{\left(\frac{t}{\delta }\right)}^{p}$	[28,41]
Three-phase linear [44]	(6) $$\left\{\begin{array}{c} Y\left(t\right)=Y_{0},t\leq \lambda \\ Y\left(t\right)=Y_{0}+\mu_{\max}\left(t-\lambda \right),\lambda <t<t_{s}\\ Y\left(t\right)=Y_{\max},\;t\geq t_{s} \end{array}\right.$$	[29,45]
Huang [46]	(7) $$\left\{\begin{array}{c} Y\left(t\right)=Y_{0}+Y_{\max}-\ln\left\{\exp\left(Y_{0}\right)+\left[\exp\left(Y_{\max}\right)-\exp\left(Y_{0}\right)\right]\exp\left(-\mu_{\max}B\left(t\right)\right)\right\}\\ B\left(t\right)=t+\frac{1}{4}\ln\frac{1+\exp\left[-4\left(t-\lambda \right)\right]}{1-\exp\left(4\lambda \right)} \end{array}\right.$$	[29,47]
No-lag phase [48]	(8) $\;Y\left(t\right)=Y_{0}+Y_{\max}-\ln\left\{\exp\left(Y_{0}\right)+\left[\exp\left(Y_{\max}\right)-\exp\left(Y_{0}\right)\right]\exp\left(-\mu_{\max}t\right)\right\}$	[47]
Net exponential	(9) $Y\left(t\right)=Y_{0}\cdot e^{\mu t}$	[49]
Modified Richards [25]	(10) $Y\left(t\right)=A{\left\{1+\nu \cdot \exp\left(1+\nu \right)\cdot \exp\left[\frac{\mu_{\max}}{A}\left(1+\nu \right)\left(1+\frac{1}{\nu }\right)\cdot \left(\lambda -t\right)\right]\right\}}^{\left(-\frac{1}{\nu }\right)}$	[29]
Modified Schnute [25]	(11) $Y\left(t\right)=\left(\mu_{\max}\frac{\left(1-b\right)}{a}\right){\left[\frac{1-b\cdot \exp\left(a\cdot \lambda +1-b-at\right)}{1-b}\right]}^{\frac{1}{b}}$	[29]
New logistic [50]	(12) $\frac{dY}{dt}=\mu_{\max}Y\left\{1-{\left(\frac{Y}{Y_{\max}}\right)}^{m}\right\}\left\{1-{\left(\frac{Y_{min}}{Y}\right)}^{n}\right\}$	[51]

**Table 2:** Systematized equations for *Vibrio* spp. secondary growth models. These models modify specific growth rate and lag time in primary models to capture effects of environmental conditions such as temperature, salinity, and pH.

Model	Equation	Article
Square root [52]	(13) $\mu_{\max}={\left[a\left(T-T_{\min}\right)\right]}^{2}$	[26,33,35,40,41,45]
Polynomial model	(14) $\lambda \;or\;\mu_{\max}=a+a_{1}T+a_{2}T^{2}+\ldots +a_{n}T^{n}$	[32,34,36,51]
Response surface [37]	(15) $$\begin{array}{c} \mu_{\max}\;or\;1/\lambda =\exp(C_{0}+C_{1}\cdot T+C_{2}\cdot a_{w}\\ +C_{3}\cdot T\cdot a_{w}+C_{4}\cdot T^{2}+C_{5}\cdot a_{w}^{2}) \end{array}$$	[37]
Arrhenius-based [23]	(16) $\mu_{\max}=\exp\left(C_{0}+\frac{C_{1}}{T}+\frac{C_{2}}{T^{2}}+C_{3}a_{w}+C_{4}a_{w}^{2}\right)$	[31,37,40,42]
Modified Ratkovsky [22]	(17) $\mu_{\max}=b{\left(T-T_{\min}\right)}^{2}\left\{1-\exp\left[c\left(T-T_{\max}\right)\right]\right\}$	[31]
Suboptimal Huang square root [53]	(18) $\mu_{\max}={\left[a{\left(T-T_{\min}\right)}^{0.75}\right]}^{2}$	[47]
Four-parameter square root and water activity [38]	(19) $$\begin{array}{c} \mu_{\max}={\left(b\left(T-T_{\min}\right)\left\{1-\exp\left[c\left(T-T_{\max}\right)\right]\right\}\right)}^{2}\;\\ \cdot \left(a_{w}-a_{w\min}\right)\left\{1-\exp\left[d\left(a_{w}-a_{w\max}\right)\right]\right\} \end{array}$$	[38]
Net *Vibrio* growth rate [49]	(20) $$\begin{array}{c} \mu_{\nu }=\left[\mu_{\max}*\mathrm{fn}\left(S,S_{\mathrm{opt}},S_{\mathrm{width}}\right)-k_{d}\right]*\theta^{T-20},\mathrm{with}\\ \;\;\mathrm{fn}\left(S,\;S_{\mathrm{opt}},S_{\mathrm{width}}\right)=\frac{-{\left(S-S_{\mathrm{opt}}\right)}^{2}}{e^{2}{\left(S_{\mathrm{width}}\right)}^{2}},\mathrm{if}\\ \;\;\;S<S_{\mathrm{opt}}-0.5\cdot S_{\mathrm{width}},\mathrm{or}\\ \;\;\;S>S_{\mathrm{opt}}-0.5\cdot S_{\mathrm{width}} \end{array}$$	[49]

**Table 3:** Parameters used in *Vibrio* spp. primary and secondary models. Last column lists models using the particular parameter.

Parameter	Description	Used in Model
Y(t) $Y_{0}$ $Y_{\max}$	Logarithm of real-time initial and maximum bacterial counts	All primary models
$\mu_{\max}$	Maximum specific growth rate	All primary models except Weibull and New logistic All secondary models except Net *Vibrio* growth rate
$\mu_{\nu }$	Net *Vibrio* growth rate	Net *Vibrio* growth rate
λ	Lag time	All primary models except Gompertz, Weibull and New logistic
t	Time	All models
$t_{s}$	Time to reach stationary growth phase	Three-phase linear
A	Maximum increase in microbial cell density	Modified logistic Gompertz Modified Gompertz Modified Richards Modified Schnute
B, D	Maximum relative growth rate and time at which the absolute growth rate is maximum	Gompertz
ν	Shape parameter	Modified Richards
a, b, c, m, n	Fitted coefficients	Modified Schnute and New logistic model
$C_{0},C_{1},C_{2}$ $C_{3},C_{4},C_{5}$	Fitted coefficients	Response surface and Arrhenius-based
T, $T_{min}$, $T_{\max}$	Temperature, minimum and maximum temperature required for growth of the organism	All secondary models
δ, p	Coefficients in the Weibull model	Weibull
$a_{w},a_{w\min},a_{w\max}$	Optimal, the minimum, and maximum water activity	Four-parameter
$S,S_{\mathrm{opt}},S_{\mathrm{width}}$	Salinity, optimal salinity value, and salinity range for optimal growth	Net *Vibrio* growth rate

**Table 4:** List of models used in the analysis. For each model, *Vibrio* spp. is specified along with the environment where the growth of the organism was observed. The primary model defines growth function and the secondary model describes functional dependencies accounting for environmental conditions (temperature, salinity, and pH). “Temp” stands for temperature, “Sal” for salinity represented in models as the concentration of NaCl (% NaCl), and “Sal (w.a.)” stands for water activity calculated from salinity. In simulations, salinity from datasets was converted to water activity when needed.

Derived Model	*Vibrio* spp.	Environment	Environmental Conditions	Primary Model	Secondary Model
Model 1 [26]	*V. cholerae*	Sea water	Temp	Modified logistic	Square root
Model 2 [26]	*V. cholerae*	Sea water	Temp	Baranyi	Square root
Model 3 [32]	*V. parahaemolyticus*	Soy sauce	Temp	Baranyi	Polynomial
Model 4 [33]	*V. parahaemolyticus*	*C. gigas*	Temp	Baranyi	Square root
Model 5 [35]	*V. parahaemolyticus*	*C. virginica*	Temp	Baranyi	Square root
Model 6 [36]	*V.* cocktail ^1^	Table Olives	pH and Sal	Baranyi	Polinomial
Model 7 [34]	*V. cholerae* and *V. vulnificus*	*O. minor*	Temp	Baranyi	Polinomial
Model 8 [37]	*V. harveyi*	TSYEB ^2^	Temp and Sal (w.a.)	Baranyi	Response surface
Model 9 [37]	*V. harveyi*	TSYEB ^2^	Temp and Sal (w.a.)	Baranyi	Arrhenius-based
Model 10 [31]	*V. parahaemolyticus*	*L. vannamei*	Temp	Baranyi	Modified Ratkowsky
Model 11 [37]	*V. harveyi*	TSYEB ^2^	Temp and Sal (w.a.)	Gompertz	Response surface
Model 12 [37]	*V. harveyi*	TSYEB ^2^	Temp and Sal (w.a.)	Gompertz	Arrhenius-based
Model 13 [38]	*V. parahaemolyticus*	Model broth system	Temp and Sal (w.a.)	Gompertz	The four-parameter square root
Model 14 [31]	*V. parahaemolyticus*	*L. vannamei*	Temp	Modified Gompertz	Modified Ratkowsky
Model 15 [40]	*V. parahaemolyticus*	Broth	Temp	Modified Gompertz	Square root Arrhenius-based
Model 16 [40]	*V. vulnificus*	Broth	Temp	Modified Gompertz	Square root Arrhenius-based
Model 17 [40]	*V. parahaemolyticus*	Flounder sashimi	Temp	Modified Gompertz	Square root Arrhenius-based
Model 18 [40]	*V. parahaemolyticus*	Salmon sashimi	Temp	Modified Gompertz	Square root Arrhenius-based
Model 19 [40]	*V. vulnificus*	Oyster meat	Temp	Modified Gompertz	Square root Arrhenius-based
Model 20 [42]	*V. parahaemolyticus* ^3^	*C. gigas* broth	Temp	Modified Gompertz	Square root Arrhenius-based
Model 21 [42]	*V. parahaemolyticus* ^4^	*C. gigas* broth	Temp	Modified Gompertz	Square root Arrhenius-based
Model 22 [42]	*V. parahaemolyticus* ^3^	*C. gigas* Oyster slurry	Temp	Modified Gompertz	Square root Arrhenius-based
Model 23 [42]	*V. parahaemolyticus* ^4^	*C. gigas* Oyster slurry	Temp	Modified Gompertz	Square root Arrhenius-based
Model 24 [41]	*V. parahaemolyticus*	*Oncorhynchus* spp.	Temp	Modified Gompertz, Weibull	Square root
Model 25 [45]	*V. parahaemolyticus*	*L. vannamei*	Temp	Three-phase linear	Square root
Model 26 [47]	*V. parahaemolyticus*	*L. vannamei*	Temp	Huang primary	Suboptimal Huang square root
Model 27 [47]	*V. parahaemolyticus*	*L. vannamei*	Temp	No-lag	Suboptimal Huang square root
Model 28 [49]	*Vibrio* spp.	NR Estuary	Temp and Sal	Net exponential	Net *Vibrio* growth rate

^1^*V. vulnificus*, *V. furnissii* and *V. fluvialis*, ^2^ Tryptone Soybean Yeast Extract Broth, ^3^ pathogenic, ^4^ nonpathogenic.

**Table 5:** The seven datasets used for model validation. AQC1 and AQC2 were previously unpublished; the other datasets are publicly available and can be accessed through the provided reference. Information for each dataset contains reported values (i.e., the number of entries in a dataset), values used for validation (i.e., the number of observations after the missing values were removed from the dataset), temperature, salinity, and pH range. Seven datasets used for model validation were classified into four habitat types based on the characteristics of the collection sites. Methods used for determining *Vibrio* spp. abundance are listed in Appendix C.1.

Dataset	Reported Values	Values for validation	Temperature Range (°C)	Salinity Range (ppt)	pH	Habitat TYPE	Collection Site
AQC1 [54]	108	99	11.1–27.5	33.5–39.3	8.10–8.61	Aquaculture	Adriatic Sea, Croatia
AQC2 [55]	88	81	7.86–25.23	24.9–38.2	7.56–8.49	Aquaculture	Adriatic Sea, Croatia
URB1 [56]	213	149	22.4–31	7.98–34.74	7.51–8.27	Urban Estuary	Ala Wai Canal in Honolulu, Hawaii
URB2 [57]	243	240	19.2–31.8	1.0–36.0	/	Urban Estuary	Ala Wai Canal in Honolulu, Hawaii
EST1 [58]	249	223	3.1–31.7	0.09–18.56	6.57–9.17	Estuary	Neuse River Estuary, North Carolina (USA)
EST2 [59]	133	127	2.16–25.89	9.32–31.86	6.82–8.41	Estuary	Great Bay Estuary, New Hampshire (USA)
COAST [60]	117	72	8.9–29.4	12.0–40.0	/	Coastal Area	Eastern North Carolina coast (USA)

**Figure A1:**
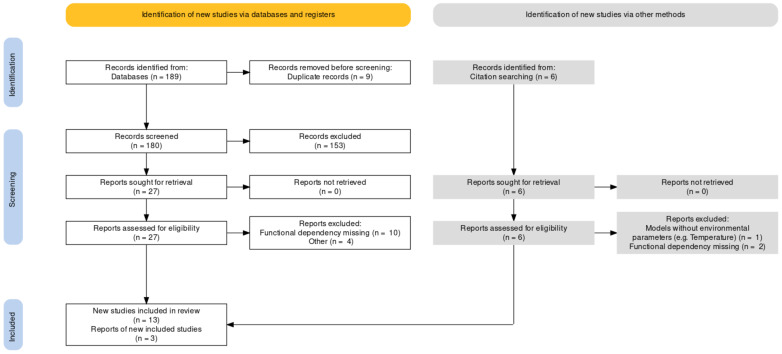
PRISMA flow chart for the literature retrieval and screening.

**Figure A2:**
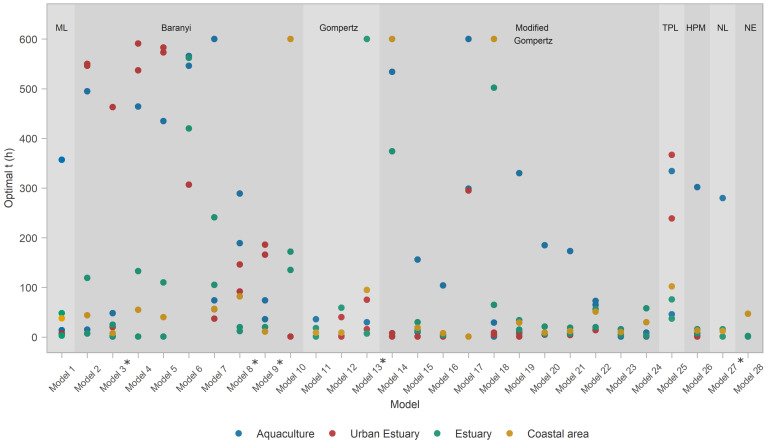
Optimal run time for the simulation duration that produces the best match between the model prediction and the observation (data point of a dataset) based on R^2^ median value. Models based on Table 4 are specified on the x axis. Primary models are labeled as follows: ML—modified logistic, Baranyi, Gompertz, modified Gompertz, TPL—three-phase linear, HPM—Huang primary, NL—no-lag, and NE—net exponential. Star (*) signifies models that had an evaluation issue with some of the data points in some of the datasets (details in Appendix D).

## Data Availability

The five datasets from literature search are available at their respective references, and the two new datasets are submitted to PANGAEA open data repository (https://www.pangaea.de/), please search for keywords AqADAPT and/or AQUAHEALTH. The code used to perform analysis and to create plots is deposited at Zenodo, https://doi.org/10.5281/zenodo.7013394 (accessed on 23 July 2022).

## Appendix A. Literature Search

The literature search was conducted using the Web of Science (WoS) advanced search in April 2022. We accessed all databases in the Web of Science: Web of Science core collection (Arts & Humanities Citation Index, Book Citation Index—Science, Book Citation Index—Social Sciences & Humanities, Conference Proceedings Citation Index—Science, Conference Proceedings Citation Index—Social Science & Humanities, Current Chemical Reactions, Emerging Sources Citation Index, ESCI Backfiles, Chemicus Index, Science Citation Index Expanded, Social Sciences Citation Index), BIOSIS citation index, Medline, Zoological record, Current contents connect, Derwent innovations index, Data citation index, SciELO citation index, BIOSIS previews, CABI–CAB abstracts and global health, Inspec, KCI–Korean journal database, journal citation reports, essential science indicators, EndNote online. The search string was defined based on keywords and Boolean and adjacency operators, and was searched for in Abstracts (Field Tag “AB”). The search string was: AB = (((vibrio*) AND (growth OR abundance) AND (temperature OR salinity OR “pH” OR “COD” OR “organic matter” OR nutrient*) AND (model*))). We obtained 189 results based on our search, which was restricted to the English language. We detected 9 potentially duplicate articles before the screening, which were resolved, and 180 remained. A primary search resulted in 27 articles for the screening. Furthermore, we performed a backward and a forward search based on identified relevant articles. A backward search was performed by searching a list of references at the end of the articles, and a forward search was conducted using Google Scholar. We performed this procedure two times until no new relevant article was identified. Finally, we included six more articles. The screening was conducted in Rayyan collaborative review application [77]. The first screening was performed by a reviewer, MP, and the final screening was carried out by reviewers TK and MP. We selected 16 papers with the *Vibrio* spp. growth models for analysis after the full screening of the papers from the primary search and additional search. Please see the PRISMA diagram for a breakdown of the overall procedure (Figure A1). In the analysis, we included articles with *Vibrio* spp. growth model equations depending on environmental parameters. Data extraction led to 28 *Vibrio* growth models for validation.

## Appendix B. Data Analysis

Data analysis was based on 28 *Vibrio* spp. growth models extracted from 16 papers that clearly defined functional dependencies for growth rate and lag time using environmental conditions (e.g., temperature, salinity, pH). For example, in Model 1, we used a modified logistic function and square root model describing the temperature; in Model 2, we used the Baranyi function and square root model describing the temperature, etc. For more details, please see Table 2 in the main text. Model 6 used the concentration of sodium chloride calculated from salinity. Model 8, Model 9, Model 11, and Model 12 used water activity determined by the concentration of sodium chloride. The water activity term, used in Model 13, was calculated from the concentration of NaCl (%), i.e., salinity. Values for water activity and the corresponding NaCl concentration (%) can be found in [78]. The function for calculating water activity from the concentration of NaCl (%) is available in R script “water_activity.R”. Analytical code for model simulations can be found in the following scripts: “AqADAPT.R”, “AQUAHEALTH.R”, “Bullington2022.R”, “Froelich2019.R”, “Steward2022.R”, “Urquhart2016.R”, and “Williams2017.R”.

**Table A1:** List of parameters used in primary model analysis. Parameter A is the maximum increase in microbial cell density, and $Y_{0}$ and $Y_{max}$ represent logarithm of initial and maximum bacterial counts, respectively. Parameter $T_{min}$ is the minimum temperature required for growth of the organism. We used the minimum value from dataset divided by 2 (Est $Y_{0}$) to estimate initial bacterial count whenever it was not provided by the authors. In cases where the maximum bacterial count was not provided, we used the estimate of the maximal bacterial count from each dataset (Est $Y_{max}$). Parameters used in secondary models are available in the provided code.

Derived Model	A	$Y_{0}$	$Y_{\max}$	$T_{\min}$ (${}^{\circ }$C)
Model 1 [26]	4	/	/	6.4 °C
Model 2 [26]	/	Est $Y_{0}$	Est $Y_{max}$	6.4
Model 3 [32]	/	Est $Y_{0}$	Est $Y_{max}$	15
Model 4 [33]	/	Est $Y_{0}$	Est $Y_{max}$	8.3
Model 5 [35]	/	Est $Y_{0}$	Est $Y_{max}$	10.0
Model 6 [36]	/	Est $Y_{0}$	Est $Y_{max}$	/
Model 7 [34]	/	Est $Y_{0}$	Est $Y_{max}$	8.0
Model 8 [37]	/	Est $Y_{0}$	Est $Y_{max}$	12.9
Model 9 [37]	/	Est $Y_{0}$	Est $Y_{max}$	12.9
Model 10 [31]	/	Est $Y_{0}$	Est $Y_{max}$	15.0
Model 11 [37]	/	Est $Y_{0}$	Est $Y_{max}$	12.9
Model 12 [37]	/	Est $Y_{0}$	Est $Y_{max}$	12.9
Model 13 [38]	/	Est $Y_{0}$	Est $Y_{max}$	8.0
Model 14 [31]	/	Est $Y_{0}$	/	15.0
Model 15 [40]	4	Est $Y_{0}$	/	13.0
Model 16 [40]	4	Est $Y_{0}$	/	13.0
Model 17 [40]	4	Est $Y_{0}$	/	13.0
Model 18 [40]	4	Est $Y_{0}$	/	13.0
Model 19 [40]	4	Est $Y_{0}$	/	13.0
Model 20 [42]	6	Est $Y_{0}$	/	10.0
Model 21 [42]	6	Est $Y_{0}$	/	10.0
Model 22 [42]	6	Est $Y_{0}$	/	10.0
Model 23 [42]	6	Est $Y_{0}$	/	10.0
Model 24 [41]	4	Est $Y_{0}$	/	12.1
Model 25 [45]	/	Est $Y_{0}$	9.28	12.1
Model 26 [47]	/	Est $Y_{0}$	7.64	10.8
Model 27 [47]	/	Est $Y_{0}$	7.70	10.5
Model 28 [49]	/	Est $Y_{0}$	/	/

## Appendix C. Additional Results

### Appendix C.1. Methods Used for Determining Vibrio spp. Abundance

In this subsection, we present more details on the techniques used to determine the abundance of *Vibrio* spp. in the studied environments. The results of different techniques (e.g., culture techniques or qPCR) are not always consistent and do not have the same meaning (e.g., possible presence of noncultivable viable bacteria). In datasets AQC1 [54] and AQC2 [55], *Vibrio* spp. abundance from water samples was determined by counting the total number of visible colonies that exhibited relief from the plate surface from the Thiosulphate Citrate Bile Salt Sucrose (TCBS) (Difco™, BD) agar plates. In the dataset URB1 [56], the polymerase chain reaction (qPCR) of the hemolysin A gene (vvhA) was used for determening *V. vulnificus* concentration in water samples. In dataset URB2 [57], the abundance of *V. vulnificus* was also determined by quantitative PCR (qPCR) of the hemolysin gene (vvhA). In dataset EST1 [58], *Vibrio* spp. concentrations were determined by counting the total number of visible yellow and green colonies that exhibited relief from the plate surface from TCBS, adjusting for dilution, and expressing the results as colony-forming units (CFUs) per 100 mL. In the dataset EST2 [59], oyster tissue was processed for enumeration of *V. parahaemolyticus* via a three-tube MPN enrichment method following the FDA Bacteriological Analytical Manual coupled with culture-based and polymerase chain reaction (PCR) methods used to confirm the presence of *V. parahaemolyticus*. In the dataset COAST [60], the authors quantified *V. parahaemolyticus* from water samples by counting the total number of visible colonies using the CHROMagar Vibrio medium (CHROMagar, Paris, France).

Model performance and its applicability to specific datasets based on a comparison of $R^{2}$ median values ($R^{2}>0.13$) resulted in a total of 27 applicable models on datasets from four habitat types (aquaculture, urban estuary, estuary, and coastal area) (Figure 2). Model 6, based on the Baranyi equation and polinomial model for the effect of pH and NaCl, exhibited poor performance. Model performance and its applicability to specific datasets based on a comparison of Q1 (upper 25%) $R^{2}$ values ($R^{2}>0.22$) resulted in a total of 21 applicable models on datasets from three areas (urban estuary, estuary, and coastal area) (Figure 2). Here, Model 3, Model 6, Model 10, Model 12, Model 17, Model 22, and Model 23 displayed poor performance. Models 2, 6, and 10 are Baranyi’s type with square root, polynomial, and modified Ratkowsky models for the effect of temperature or pH and NaCl, respectively. Model 12 is Gompertz’s type with modified Ratkovsky and Arrhenius-based models, respectively. Model 22 and Model 23 were based on the modified Gompertz model and used square root and Arrhenius-based models for describing the effect of temperature.

**Table A2:** Results of robust ANOVA one-way test from the package WRS2 [61].

Function:	t1way (formula = max_r2~Habitat, data = as)
Test statistic:	F = 75.561
Degrees of freedom 1:	3
Degrees of freedom 2:	49.90
p-value:	0
Explanatory measure of effect size:	0.77
Bootstrap CI:	[0.68; 0.84]

**Table A3:** Results of post hoc lincon test from the package WRS2 [61].

Formula:		lincon (max_r2~Habitat, data = as)		
Habitat type	psihat	ci.lower	ci. upper	*p*-value
Aquaculture vs. Urban Estuary	−0.03910	−0.08237	0.00417	0.01688
Aquaculture vs. Estuary	−0.14203	−0.17331	−0.11075	0.00000
Aquaculture vs. Coastal Area	−0.21525	−0.27062	−0.15989	0.00000
Urban Estuary vs. Estuary	−0.10293	−0.14925	−0.05662	0.00000
Urban Estuary vs. Coastal Area	−0.17616	−0.23977	−0.11254	0.00000
Estuary vs. Coastal Area	−0.07322	−0.13069	−0.01575	0.00263

## Appendix D. Model Evaluation Issues

Some models had evaluation issues, where a proportion of the data had to be disregarded. The issues for each model are described below, and Table A4 summarizes the issues and proportion of data disregarded at the optimum simulation time.

Model 3 had negative specific growth rates generated by the secondary model (the parameters used for a four-parameter polynomial model by [32]). This phenomenon was observed for temperatures below 15 °C. Model 3 also generated Inf values for higher simulation times and higher specific growth rates. Model 7 generated negative specific growth rates for temperatures between 11.1 and 11.7 °C. A secondary model was polynomial, as described in [34]. Additionally, in this secondary model, lag time had lower values at 17.6 °C and the highest at 27.5 °C. Model 8 resulted in Inf values in cases where maximum specific growth rate was between 1.630371 (12.3 °C) and 2.476982 (27.5 °C) and time range was between 289 and 600 h. This large value of the specific growth rate was generated by the response surface secondary model from [37]. Some of the predicted values were equal to the parameter maximum bacterial count ($Y_{max}$) (we obtained NA $R^{2}$). Model 9 resulted in Inf values in cases where mumax was between 1.491716 (15.9 °C) and 2.459676 (27.5 °C) and the time range was between 291 and 600. This large value of the specific growth rate was generated by Arrhenius–Davey secondary model from [37]. Some of the predicted values were equal to the parameter maximum bacterial count ($Y_{max}$) (we obtained NA $R^{2}$). Model 13 resulted in NAN predictions for values that had salinity, i.e., water activity higher than 0.998, and Model 27 had NAN predictions for temperatures below 10.5 °C.

**Table A4:** Models and their issues.

Model	Issue	Impacts % of Dataset	Dataset
Model 3	Negative specific growth rate	26/99 = 26.26%	AQC1 [54]
		16/81 = 19.75%	AQC2 [55]
		52/223 = 23.32%	EST1 [58]
		30/127 = 23.62%	EST2 [59]
		4/72 = 5.56%	COAST [60]
Model 8	High values of specific growth rate which generate Inf values	/	/
Model 9	High values of specific growth rate which generate Inf values	1/81 = 1.23%	AQC2 [55]
		6/223 = 2.69%	EST1 [58]
		4/127 = 3.15%	EST2 [59]
Model 13	Salinity, i.e., water activity >0.998	80/223 = 35.87%	EST1 [58]
		18/240 = 7.50%	URB2 [57]
Model 27	Temperature 10.5 °C	2/81 = 2.47%	AQC2 [55]
		29/223 = 13.00%	EST1 [58]
		16/127 = 12.60%	EST2 [59]
		1/72 = 1.39%	COAST [60]

## References

[1] Zhang X., Lin H., Wang X., Austin B. (2018). Significance of Vibrio species in the marine organic carbon cycle—A review. Sci. China Earth Sci..

[2] Sampaio A., Silva V., Poeta P., Aonofriesei F. (2022). *Vibrio* spp.: Life strategies, ecology, and risks in a changing environment. Diversity.

[3] Thompson J.R., Polz M.F. (2006). Dynamics of Vibrio populations and their role in environmental nutrient cycling. The Biology of Vibrios.

[4] Beardsley C., Pernthaler J., Wosniok W., Amann R. (2003). Are readily culturable bacteria in coastal North Sea waters suppressed by selective grazing mortality?. Appl. Environ. Microbiol..

[5] Ganesan D., Gupta S.S., Legros D. (2020). Cholera surveillance and estimation of burden of cholera. Vaccine.

[6] Gilbert J.A., Steele J.A., Caporaso J.G., Steinbrück L., Reeder J., Temperton B., Huse S., McHardy A.C., Knight R., Joint I. (2012). Defining seasonal marine microbial community dynamics. ISME J..

[7] Westrich J.R., Ebling A.M., Landing W.M., Joyner J.L., Kemp K.M., Griffin D.W., Lipp E.K. (2016). Saharan dust nutrients promote Vibrio bloom formation in marine surface waters. Proc. Natl. Acad. Sci. USA.

[8] Poloczanska E.S., Brown C.J., Sydeman W.J., Kiessling W., Schoeman D.S., Moore P.J., Brander K., Bruno J.F., Buckley L.B., Burrows M.T. (2013). Global imprint of climate change on marine life. Nat. Clim. Chang..

[9] Dore M.H. (2005). Climate change and changes in global precipitation patterns: What do we know?. Environ. Int..

[10] Bijma J., Pörtner H.O., Yesson C., Rogers A.D. (2013). Climate change and the oceans—What does the future hold?. Mar. Pollut. Bull..

[11] Burge C.A., Hershberger P.K., Behringer D.C., Silliman B.R., Lafferty K.D. (2020). Climate change can drive marine diseases. Marine Disease Ecology.

[12] Martinez-Urtaza J., Bowers J.C., Trinanes J., DePaola A. (2010). Climate anomalies and the increasing risk of Vibrio parahaemolyticus and Vibrio vulnificus illnesses. Food Res. Int..

[13] Da Cunha D.T. (2021). Improving food safety practices in the foodservice industry. Curr. Opin. Food Sci..

[14] Levallois P., Villanueva C.M. (2019). Drinking water quality and human health: An editorial. Int. J. Environ. Res. Public Health.

[15] Li H., Xie G., Edmondson A.S. (2008). Review of secondary mathematical models of predictive microbiology. J. Food Prod. Mark..

[16] Ferrer J., Prats C., López D., Vives-Rego J. (2009). Mathematical modeling methodologies in predictive food microbiology: A SWOT analysis. Int. J. Food Microbiol..

[17] Peleg M., Corradini M.G. (2011). Microbial growth curves: What the models tell us and what they cannot. Crit. Rev. Food Sci. Nutr..

[18] Perez-Rodriguez F., Valero A. (2013). Predictive microbiology in foods. Predictive Microbiology in Foods.

[19] Oscar T. (2005). Development and validation of primary, secondary, and tertiary models for growth of Salmonella Typhimurium on sterile chicken. J. Food Prot..

[20] Baranyi J., McClure P., Sutherland J., Roberts T. (1993). Modeling bacterial growth responses. J. Ind. Microbiol. Biotechnol..

[21] Pirt S.J. (1975). Principles of Microbe and Cell Cultivation.

[22] Ratkowsky D., Lowry R., McMeekin T., Stokes A., Chandler R. (1983). Model for bacterial culture growth rate throughout the entire biokinetic temperature range. J. Bacteriol..

[23] Davey K. (1989). A predictive model for combined temperature and water activity on microbial growth during the growth phase. J. Appl. Bacteriol..

[24] Esser D.S., Leveau J.H., Meyer K.M. (2015). Modeling microbial growth and dynamics. Appl. Microbiol. Biotechnol..

[25] Zwietering M., Jongenburger I., Rombouts F., Van’t Riet K. (1990). Modeling of the bacterial growth curve. Appl. Environ. Microbiol..

[26] Fu S., Shen J., Liu Y., Chen K. (2013). A predictive model of V ibrio cholerae for combined temperature and organic nutrient in aquatic environments. J. Appl. Microbiol..

[27] Fu S., Liu Y., Li X., Tu J., Lan R., Tian H. (2015). A preliminary stochastic model for managing microorganisms in a recirculating aquaculture system. Ann. Microbiol..

[28] Ma F., Liu H., Wang J., Zhang Z., Sun X., Pan Y., Zhao Y. (2016). Behavior of Vibrio parahemolyticus cocktail including pathogenic and nonpathogenic strains on cooked shrimp. Food Control.

[29] Zahaba M., Halmi M.I.E., Othman A.R., Shukor M.Y. (2018). Mathematical modeling of the growth curve of Vibrio sp. isolate MZ grown in Seawater medium. Bioremediation Sci. Technol. Res..

[30] Baranyi J., Roberts T.A. (1995). Mathematics of predictive food microbiology. Int. J. Food Microbiol..

[31] Boonyawantang A., Mahakarnchanakul W., Rachtanapun C., Boonsupthip W. (2012). Behavior of pathogenic Vibrio parahaemolyticus in prawn in response to temperature in laboratory and factory. Food Control.

[32] Chung K.H., Park M.S., Kim H.Y., Bahk G.J. (2019). Growth prediction and time–temperature criteria model of Vibrio parahaemolyticus on traditional Korean raw crab marinated in soy sauce (ganjang-gejang) at different storage temperatures. Food Control.

[33] Fernandez-Piquer J., Bowman J.P., Ross T., Tamplin M.L. (2011). Predictive models for the effect of storage temperature on Vibrio parahaemolyticus viability and counts of total viable bacteria in Pacific oysters (Crassostrea gigas). Appl. Environ. Microbiol..

[34] Oh H., Yoon Y., Ha J., Lee J., Shin I.S., Kim Y.M., Park K.S., Kim S. (2021). Risk assessment of vibriosis by Vibrio cholerae and Vibrio vulnificus in whip-arm octopus consumption in South Korea. Fish. Aquat. Sci..

[35] Parveen S., DaSilva L., DePaola A., Bowers J., White C., Munasinghe K.A., Brohawn K., Mudoh M., Tamplin M. (2013). Development and validation of a predictive model for the growth of Vibrio parahaemolyticus in post-harvest shellstock oysters. Int. J. Food Microbiol..

[36] Posada-Izquierdo G.D., Valero A., Arroyo-López F.N., González-Serrano M., Ramos-Benítez A.M., Benítez-Cabello A., Rodríguez-Gómez F., Jimenez-Diaz R., García-Gimeno R.M. (2021). Behavior of *Vibrio* spp. in table olives. Front. Microbiol..

[37] Zhou K., Gui M., Li P., Xing S., Cui T., Peng Z. (2012). Effect of combined function of temperature and water activity on the growth of Vibrio harveyi. Braz. J. Microbiol..

[38] Miles D.W., Ross T., Olley J., McMeekin T.A. (1997). Development and evaluation of a predictive model for the effect of temperature and water activity on the growth rate of Vibrio parahaemolyticus. Int. J. Food Microbiol..

[39] Nishina T., Wada M., Ozawa H., Hara-Kudo Y., Konuma H., Hasegawa J., Kumagi S. (2004). Growth kinetics of Vibrio parahaemolyticus O3: K6 under varying conditions of pH, NaCl concentration and temperature. Food Hyg. Saf. Sci..

[40] Kim Y.W., Lee S.H., Hwang I.G., Yoon K.S. (2012). Effect of temperature on growth of Vibrio paraphemolyticus and Vibrio vulnificus in flounder, salmon sashimi and oyster meat. Int. J. Environ. Res. Public Health.

[41] Yang Z.Q., Jiao X.A., Li P., Pan Z.M., Huang J.L., Gu R.X., Fang W.M., Chao G.X. (2009). Predictive model of Vibrio parahaemolyticus growth and survival on salmon meat as a function of temperature. Food Microbiol..

[42] Yoon K., Min K., Jung Y., Kwon K., Lee J., Oh S. (2008). A model of the effect of temperature on the growth of pathogenic and nonpathogenic Vibrio parahaemolyticus isolated from oysters in Korea. Food Microbiol..

[43] Van Boekel M.A. (2002). On the use of the Weibull model to describe thermal inactivation of microbial vegetative cells. Int. J. Food Microbiol..

[44] Buchanan R., Whiting R., Damert W. (1997). When is simple good enough: A comparison of the Gompertz, Baranyi, and three-phase linear models for fitting bacterial growth curves. Food Microbiol..

[45] Tang X., Zhao Y., Sun X., Xie J., Pan Y., Malakar P.K. (2015). Predictive model of Vibrio parahaemolyticus O3: K6 growth on cooked Litopenaeus vannamei. Ann. Microbiol..

[46] Huang L. (2008). Growth kinetics of Listeria monocytogenes in broth and beef frankfurters—Determination of lag phase duration and exponential growth rate under isothermal conditions. J. Food Sci..

[47] Chen Y.R., Hwang C.A., Huang L., Wu V.C., Hsiao H.I. (2019). Kinetic analysis and dynamic prediction of growth of vibrio parahaemolyticus in raw white shrimp at refrigerated and abuse temperatures. Food Control.

[48] Fang T., Gurtler J.B., Huang L. (2012). Growth kinetics and model comparison of Cronobacter sakazakii in reconstituted powdered infant formula. J. Food Sci..

[49] Froelich B., Bowen J., Gonzalez R., Snedeker A., Noble R. (2013). Mechanistic and statistical models of total Vibrio abundance in the Neuse River Estuary. Water Res..

[50] Fujikawa H., Kai A., Morozumi S. (2003). A new logistic model for bacterial growth. Shokuhin Eiseigaku Zasshi. J. Food Hyg. Soc. Jpn..

[51] Fujikawa H. (2011). Application of the new logistic model to microbial growth prediction in food. Biocontrol Sci..

[52] Ratkowsky D.A., Olley J., McMeekin T., Ball A. (1982). Relationship between temperature and growth rate of bacterial cultures. J. Bacteriol..

[53] Huang L., Hwang C.A., Phillips J. (2011). Evaluating the Effect of Temperature on Microbial Growth Rate—The Ratkowsky and a Bělehrádek-Type Models. J. Food Sci..

[54] Kapetanović D., Purgar M., Gavrilović A., Hackenberger B.K., Kurtović B., Haberle I., Pečar Ilić J., Geček S., Hackenberger D.K., Djerdj T. (2022). AqADAPT: Physiochemical parameters and *Vibrio* spp. abundance in water samples gathered in the Adriatic Sea. PANGAEA.

[55] Jug-Dujaković J., Gavrilović A., Kolda A., Žunić J., Pikelj K., Listeš E., Vukić Lušić D., Vardić Smrzlić L., Perić L., El-Matbouli M. (2022). AQUAHEALTH: Physiochemical parameters and *Vibrio* spp. abundance in water samples gathered in the Adriatic Sea. PANGAEA.

[56] Bullington J.A., Golder A.R., Steward G.F., McManus M.A., Neuheimer A.B., Glazer B.T., Nigro O.D., Nelson C.E. (2022). Refining real-time predictions of Vibrio vulnificus concentrations in a tropical urban estuary by incorporating dissolved organic matter dynamics. Sci. Total Environ..

[57] Nigro O.D., James-Davis L.I., De Carlo E.H., Li Y.H., Steward G.F. (2022). Variable freshwater influences on the abundance of Vibrio vulnificus in a tropical urban estuary. Appl. Environ. Microbiol..

[58] Froelich B., Gonzalez R., Blackwood D., Lauer K., Noble R. (2019). Decadal monitoring reveals an increase in *Vibrio* spp. concentrations in the Neuse River Estuary, North Carolina, USA. PLoS ONE.

[59] Urquhart E.A., Jones S.H., Yu J.W., Schuster B.M., Marcinkiewicz A.L., Whistler C.A., Cooper V.S. (2016). Environmental conditions associated with elevated Vibrio parahaemolyticus concentrations in Great Bay Estuary, New Hampshire. PLoS ONE.

[60] Williams T.C., Froelich B.A., Phippen B., Fowler P., Noble R.T., Oliver J.D. (2017). Different abundance and correlational patterns exist between total and presumed pathogenic Vibrio vulnificus and V. parahaemolyticus in shellfish and waters along the North Carolina coast. FEMS Microbiol. Ecol..

[61] Mair P., Wilcox R. (2020). Robust statistical methods in R using the WRS2 package. Behav. Res. Methods.

[62] Wilcox R.R. (2012). Introduction to Robust Estimation and Hypothesis Testing.

[63] RStudio Team (2020). RStudio: Integrated Development Environment for R.

[64] Wickham H., Averick M., Bryan J., Chang W., McGowan L.D., François R., Grolemund G., Hayes A., Henry L., Hester J. (2019). Welcome to the tidyverse. J. Open Source Softw..

[65] Kuhn M. (2008). Building Predictive Models in R Using the caret Package. J. Stat. Software Artic..

[66] Hamner B., Frasco M., LeDell E. (2018). Evaluation Metrics for Machine Learning. https://cran.r-project.org/web/packages/Metrics/Metrics.pdf.

[67] Grosjean P. (2019). SciViews–Main Package. https://cran.r-project.org/web/packages/SciViews/SciViews.pdf.

[68] Dowle M. (2021). Package ‘data.table’: Extension of ‘data.frame’. https://cran.r-project.org/web/packages/data.table/data.table.pdf.

[69] Wickham H. (2019). Read Excel Files. https://cran.microsoft.com/snapshot/2019-03-09/web/packages/readxl/readxl.pdf.

[70] Dragulescu A., Arendt C. (2020). Read, Write, Format Excel 2007 and Excel 97/2000/XP/2003 Files. https://cran.r-project.org/web/packages/xlsx/xlsx.pdf.

[71] Purgar M., Kapetanović D., Geček S., Marn N., Haberle I., Hackenberger K.B., Gavrilović A., Pečar Ilić J., Hackenberger K. D., Djerdj T. (2022). Codes for Purgar et al. 2022: Investigating the ability of growth models to predict in situ *Vibrio* spp. abundances. Zenodo.

[72] Wickham H. (2016). ggplot2: Elegant Graphics for Data Analysis.

[73] Martinez-Urtaza J., Lozano-Leon A., Varela-Pet J., Trinanes J., Pazos Y., Garcia-Martin O. (2008). Environmental determinants of the occurrence and distribution of Vibrio parahaemolyticus in the rias of Galicia, Spain. Appl. Environ. Microbiol..

[74] Kirchman D.L. (2016). Growth rates of microbes in the oceans. Annu. Rev. Mar. Sci..

[75] Norris N., Levine N.M., Fernandez V.I., Stocker R. (2021). Mechanistic model of nutrient uptake explains dichotomy between marine oligotrophic and copiotrophic bacteria. PLoS Comput. Biol..

[76] Trinanes J., Martinez-Urtaza J. (2021). Future scenarios of risk of Vibrio infections in a warming planet: A global mapping study. Lancet Planet. Health.

[77] Ouzzani M., Hammady H., Fedorowicz Z., Elmagarmid A. (2016). Rayyan—A web and mobile app for systematic reviews. Syst. Rev..

[78] Gekas V., Gonzalez C., Sereno A., Chiralt A., Fito P. (1998). Mass transfer properties of osmotic solutions. I. Water activity and osmotic pressure. Int. J. Food Prop..

